# Water consumption, grain yield, and water productivity in response to field water management in double rice systems in China

**DOI:** 10.1371/journal.pone.0189280

**Published:** 2017-12-07

**Authors:** Xiao Hong Wu, Wei Wang, Chun Mei Yin, Hai Jun Hou, Ke Jun Xie, Xiao Li Xie

**Affiliations:** 1 Faculty of Life Science and Technology, Central South University of Forestry and Technology, Changsha, Hunan, China; 2 Key Laboratory of Agro-ecological Processes in Subtropical Region, Institute of Subtropical Agriculture, Chinese Academy of Sciences, Changsha, Hunan, China; 3 Hunan Agricultural Resources and Environmental Protection Management Station, Changsha, Hunan, China; University of Vigo, SPAIN

## Abstract

Rice cultivation has been challenged by increasing food demand and water scarcity. We examined the responses of water use, grain yield, and water productivity to various modes of field water managements in Chinese double rice systems. Four treatments were studied in a long-term field experiment (1998–2015): continuous flooding (CF), flooding—midseason drying—flooding (F-D-F), flooding—midseason drying—intermittent irrigation without obvious standing water (F-D-S), and flooding—rain-fed (F-RF). The average precipitation was 483 mm in early-rice season and 397 mm in late-rice season. The irrigated water for CF, F-D-F, F-D-S, and F-RF, respectively, was 263, 340, 279, and 170 mm in early-rice season, and 484, 528, 422, and 206 mm in late-rice season. Grain yield for CF, F-D-F, F-D-S, and F-RF, respectively, was 4,722, 4,597, 4,479, and 4,232 kgha^-1^ in early-rice season, and 5,420, 5,402, 5,366, and 4,498 kgha^-1^ in late-rice season. Compared with CF, F-D-F consumed more irrigated water, which still decreased grain yield, leading to a decrease in water productivity by 25% in early-rice season and by 8% in late-rice season. Compared with F-D-F, F-D-S saved much irrigated water with a small yield reduction, leading to an increase in water productivity by 22% in early-rice season and by 26% in late-rice season. The results indicate that CF is best for early-rice and FDS is best for late-rice in terms of grain yield and water productivity.

## Introduction

Rice (*Oryza sativa L*.) is planted annually on areas of about 154 million hectares, taking up about 11% of the world’s cultivated land [1]. In fact, 90% of rice is grown in Asia, which consumes about 80% of the total irrigated fresh water resources around the world [1–2]. Water for agricultural use becomes increasingly scarce due to climate change and rapid industrialization and urbanization [3–5]. By the year 2025, irrigated rice of 15–20 million hectares in Asia will suffer water scarcity [6]. Farmers are facing a challenge to produce more rice per unit land with limited water in order to meet the food demand of the growing population. This is crucial for food security in many Asian countries where large and dense populations depend on subsistence agriculture [7–10]. China is an important rice producer in Asia. From 2004 to 2014, the total rice production in China increased from 180 million tons to 208 million tons, with per unit area yield increasing from 6,308 kg ha^-1^ in 2004 to 6,811 kg ha^-1^ in 2014. However, the population in China has increased from 1.36 billion to 1.44 billion during the same period.

Water is essential for growth and development of rice plants. However, continuous flooding results in a large amount of unproductive water outflows through evaporation, seepage, and percolation [11–13]. Growing evidence indicates that continuous flooding is unnecessary for rice to achieve high yields, which, however, is based on short-term trials. Long-term field water conditions would produce profound changes in soil properties, which may further affect soil water conservation and crop yield. However, few studies have been conducted on long-term field trial. So, there is little information on water consumption, crop yield, and water productivity after long-term adoption of water-saving irrigation.

Rice agriculture occupies 23% of cultivated land in China [14], mostly distributed in the south. Since fresh water distribution is distributed unevenly both spatially and temporally, most farmers try to reserve rainwater in the field as much as possible unless when significantly negative impacts occurred due to deep water. When the soil dries to a certain threshold, farmers begin to irrigate the soil so that it is flooded or saturated. Rice is also grown traditionally under rain-fed conditions, mainly due to lack of access to irrigation. Currently, typical modes of water management include continuous flooding, flooding—midseason drying—flooding, flooding—midseason drying—intermittent irrigation, and flooding—rain-fed, among which flooding—midseason drying—flooding is the most popular with farmers. This study was carried out on a long-term water management experiment field in southern China, which was initiated in 1998. The objective of this study is to quantify the impact of long-term water management on water consumption, crop yield, and water productivity in a red clay soil under the climatic conditions in south China.

## Materials and methods

### Experiment site

This study was carried out at Taoyuan Station of Agro-ecology Research (111°27′ E, 28°55′ N; altitude: 92.2–125.3 m), Hunan province, China. The region is characterized by the subtropical humid monsoon climate, with an annual average air temperature of 16.5°C, precipitation of 1,448 mm, sunshine of 1,513 h, and frost-free period of 283 days. Frost generally occurred in December, January, and February. Transplanted double rice generally grow from late April to October, with a minimum daily air temperature at around 20°C, a maximum daily air temperature at around 31°C, and sunshine of 950 h. The soil here was Stagnic Anthrosols developed from Quaternary red clay. The topsoil (0~20 cm, in 1998) properties: pH 5.7, bulk density 1.03 g cm^−3^, organic C 12.8 g kg^–1^, total N 1.45 g kg^–1^, and total P 0.53 g kg^–1^.

### Experimental design

The experimental plots have been established since 1998. The modes of water management included continuous flooding (CF), flooding—midseason drying—flooding (F-D-F), flooding—midseason drying—intermittent irrigation without obvious standing water (F-D-S), and flooding—rain-fed (F-RF). Each treatment had three replicates. Each plot was 6.2 m × 6.2 m in size. Plots were separated by a 15 cm wide cement wall which was buried into soil to a depth of 150 cm, with a height of 20 cm above soil.

### Water management

In all treatments, the fields were flooded with a water layer of about 10 cm for land preparation and seeding transplanting. Midseason drying was carried out at the end of tillering stage, about 25 days and 30 days after transplanting for early-rice and late-rice, respectively. During the several days before midseason drying, shallow water was kept to avoid redundant irrigation. In the CF plots, irrigation was carried out to increase the water layer to a depth of 10 cm when the water layer decreased to a depth of 2 cm. In the F-D-F plots, the water layer was replenished to 10 cm when water layer decreased to a depth of 2 cm after midseason drying. In the F-D-S plots, the soil was saturated with no obvious standing water by intermittent irrigation when water table decreased to 3 cm below soil surface after midseason drying. In the F-RF plots, the fields would not be irrigated since 15 days after transplanting, so the soil moisture was often below saturation from shooting stage to harvest, especially in late-rice seasons. CF collected rain water while the other three treatments implemented drainage during fallow season.

### Crop management

Varieties used in early-rice season included *xiangzaoxian21* (1998), *zhongqian100* (1999–2001), *xiangzaoxian32* (2002–2004), *xiangzaoxian24* (2002–2007), *xiangzaoxian25* (2008), *xiangzaoxian43* (2009), *xiangzaoxian44* (2010), *xiangzaoxian44* (2011–2012), and z*hongzao39* (2013–2015). Varieties used in late-rice season included *xianyougui99* (1998–1999), *jinyougui99* (2000–2001), *xianyou46* (2002–2004), *jinyou207* (2005–2008), *T-you207* (2009), *fengyuanyou299* (2010–2011), *shenyou9586* (2012), and *fengyuanyou277* (2013–2015). Generally, early-rice season was from late April to mid July, and late-rice season was from mid July to mid October. The fertilizers applied were urea for nitrogen (N), calcium superphosphate for phosphorus (P), and potassium chloride for potassium (K). Early-rice season received 81 kg N ha^-1^ (50% as basal fertilizer and 50% as tillering stage fertilizer), 39.3 kg P ha^-1^ (as basal fertilizer), and 88 kg K ha^-1^ (as basal fertilizer). Late-rice season received 101 kg N ha^-1^ with three splits, (50% as basal fertilizer, 33% as tillering stage fertilizer and 17% as panicle fertilizer) and 110 kg K ha^-1^ (as basal fertilizer). Weeds, insects, and diseases were controlled following the local practices.

### Observation

Precipitation and evaporation were measured at a weather station nearby the experiment field, which was within 100 meters. The volume of irrigated water was monitored with a water flow meter installed in the irrigation pipeline. The amount of irrigated water (mm) was calculated as volume of irrigated water divided by plot area. The rice plants at maturity stage in each plot were hand harvested. Grain samples were oven-dried at 70°C and weighed. The grain yield was determined on the basis of 140 g kg^-1^ water content. Water productivity (kg m^-3^) was calculated as grain yield (kg ha^-1^) divided by total amount of irrigated water (mm).

### Statistical analysis

Statistical analyses were performed with SPSS 17.0 (SPSS, Inc., USA). Multiple comparisons of significant differences were made using Duncan’s test (*P* < 0.05). Correlation analyses were performed using Pearson correlation analysis.

## Results

### Effect of seasonal conditions on crop performance

**Fig 1:**
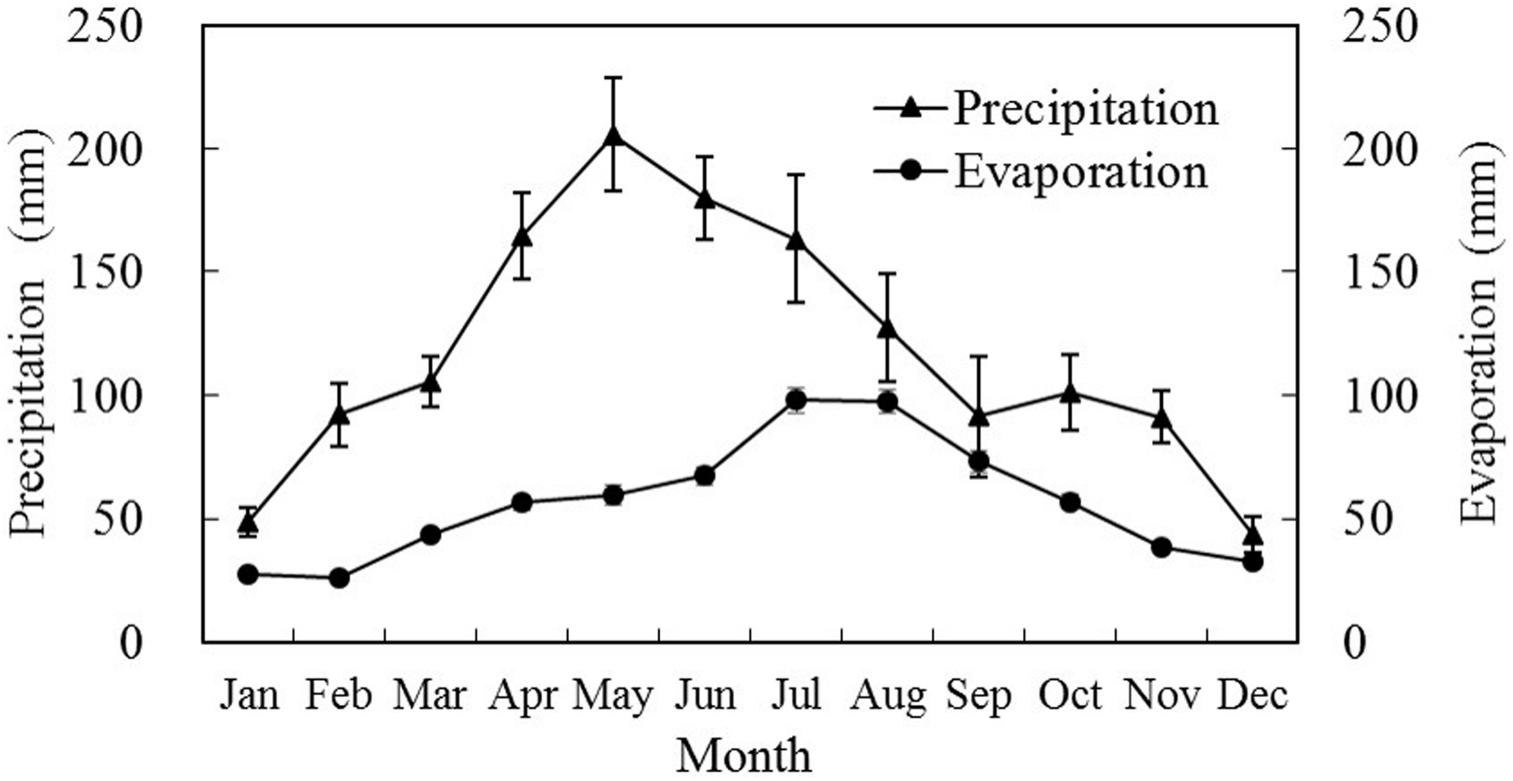
Monthly precipitation and evaporation in experimental site during 1998–2015.

According to field observation results from 1998–2015 at the experimental site, the annual average precipitation was 1,414 mm, which was concentrated between April and July, accounting for around 50% of the total precipitation through the year (Fig 1). It could basically meet the water demand of the growth of early-rice. Annual amount of evaporation was 676 mm, accounting for 48% of annual precipitation. The evaporation occurred intensively between June and September (336 mm), accounting for 50% of annual evaporation. From August to September, the total precipitation was low, which as 219 mm, and the total evaporation was high, which reached 170 mm. This period, however, is a critical stage for the growth of late-rice, indicating that large amount of irrigation is inevitable. At the same time, the precipitation varied greatly during this period, with annual variable coefficient being 24% and the variable coefficient between August and September reaching 61%. The early-rice season coincides with the rainy season, which allows successful early-rice cultivation with less irrigation. In contrast, less precipitation occurred in late-rice season, suggesting more irrigation for late-rice.

### Water consumption

**Fig 2:**
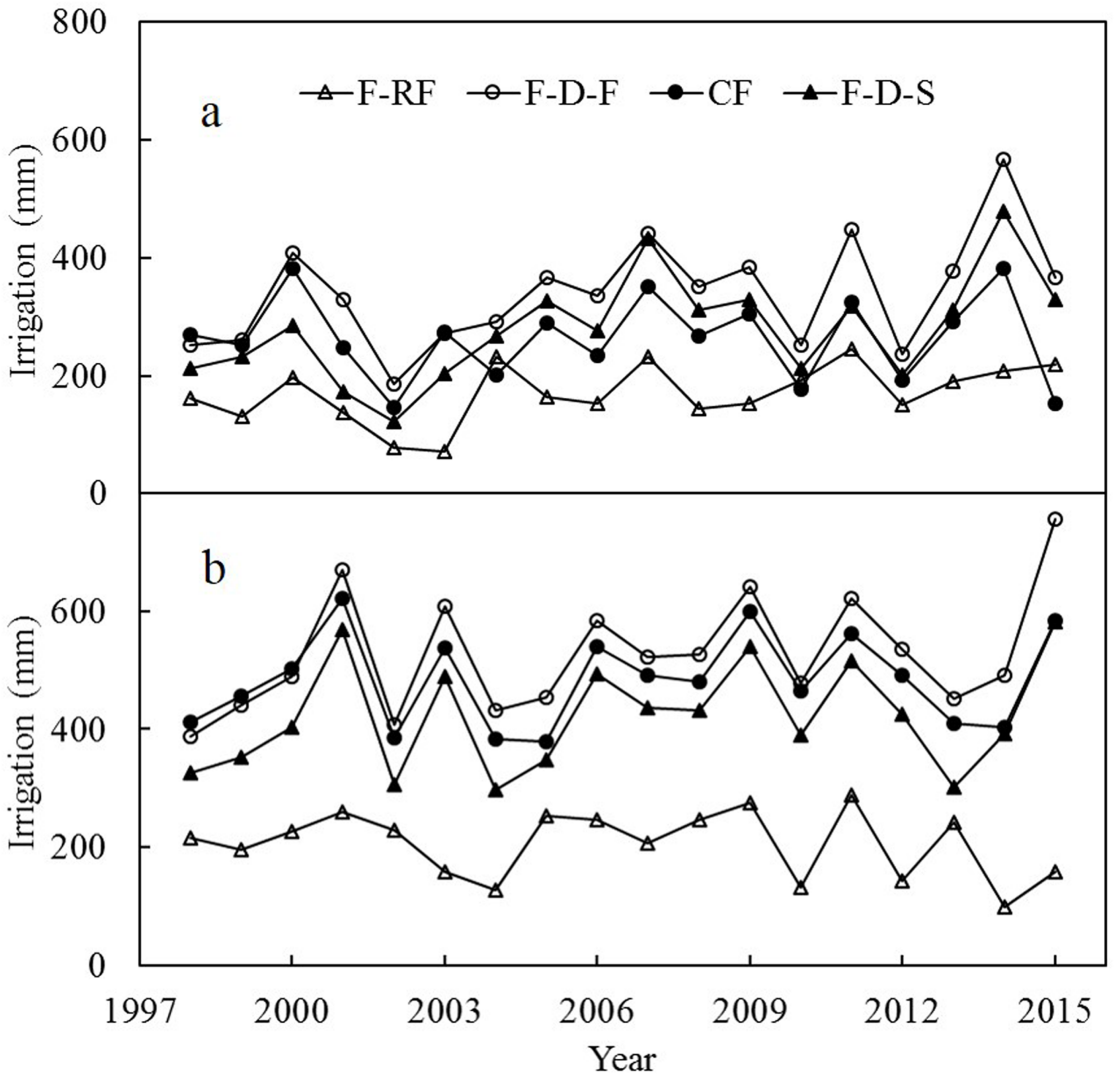
Irrigated water in early-rice season (a) and late-rice season (b) under different treatments. CF, continuous flooding; F-D-F, flooding—midseason drying—flooding; F-D-S, flooding—midseason drying—intermittent irrigation without obvious standing water; F-RF, flooding—rain-fed.

Irrigated water under different modes of water management is presented in Fig 2 and Table 1. From 1998–2015, irrigated water for F-D-F, F-D-S, CF, and F-RF, respectively, ranged from 186–566 mm, 121–478 mm, 146–383 mm, and 72–246 mm in early-rice season; and ranged from 388–755 mm, 298–581 mm, 379–621 mm, and 100–289 mm in late-rice season (Fig 2). On average, the ranking of irrigated water was F-D-F > F-D-S > CF > F-RF in early-rice season and F-D-F > CF > F-D-S > F-RF in late-rice season (Table 1). Compared with CF, F-D-F increased irrigated water by 29% in early-rice season and 9% in late-rice season. F-D-S increased irrigated water by 6% in early-rice season and reduced irrigated water by 13% in late-rice season. F-RF reduced irrigated water by 35% in early-rice season and 57% in late-rice season. In early-rice season, irrigated water in the first several years in CF plots was roughly equal to that in F-D-F plots, and more than that in F-D-S plots. After that, irrigated water in CF plots was less than that in F-D-F and F-D-S plots in general. The precipitation was 483 mm in early-rice season and 397 mm in late-rice season on average during 1998–2015. Irrigated water was negatively correlated with precipitation (Table 2).

**Table 1:** Annual mean values of irrigated water, grain yield, and water productivity (WP) under different treatments during 1998–2015.

Treatments	Early-rice season			Late-rice season		
	Irrigated water mm	Grain yield kg ha^-1^	WP kg m^-3^	Irrigated water mm	Grain yield kg ha^-1^	WP kg m^-3^
**F-D-F**	340 a	4597 a	1.42 b	528 a	5402 a	1.05 b
**CF**	263 b	4722 a	1.90 b	484 a	5420 a	1.14 b
**F-D-S**	279 b	4479 a	1.73 b	422 b	5366 a	1.32 b
**F-RF**	170 c	4232 a	2.75 a	206 c	4498 b	2.40 a

Different letters, per column, indicate significantly difference using Duncan’s test (*P <* 0.05). See Fig 2 for CF, F-D-F, F-D-S, and F-RF.

**Table 2:** Correlations between irrigated water and precipitation under different treatments during 1998–2015.

	Precipitation	
	Early rice season	Late rice season
**Irrigated water in F-D-F**	*r* = -0.792, *P* < 0.001	*r* = -0.898, *P* < 0.001
**Irrigated water in CF**	*r* = -0.625, *P* = 0.006	*r* = -0.828, *P* < 0.001
**Irrigated water in F-D-S**	*r* = -0.703, *P* = 0.001	*r* = -0.911, *P* < 0.001
**Irrigated water in F-RF**	*r* = -0.587, *P* = 0.011	*r* = -0.268, *P* = 0.281

Correlation analyses were performed using Pearson correlation analysis. See Fig 2 for CF, F-D-F, F-D-S, and F-RF.

### Rice productivity

**Fig 3:**
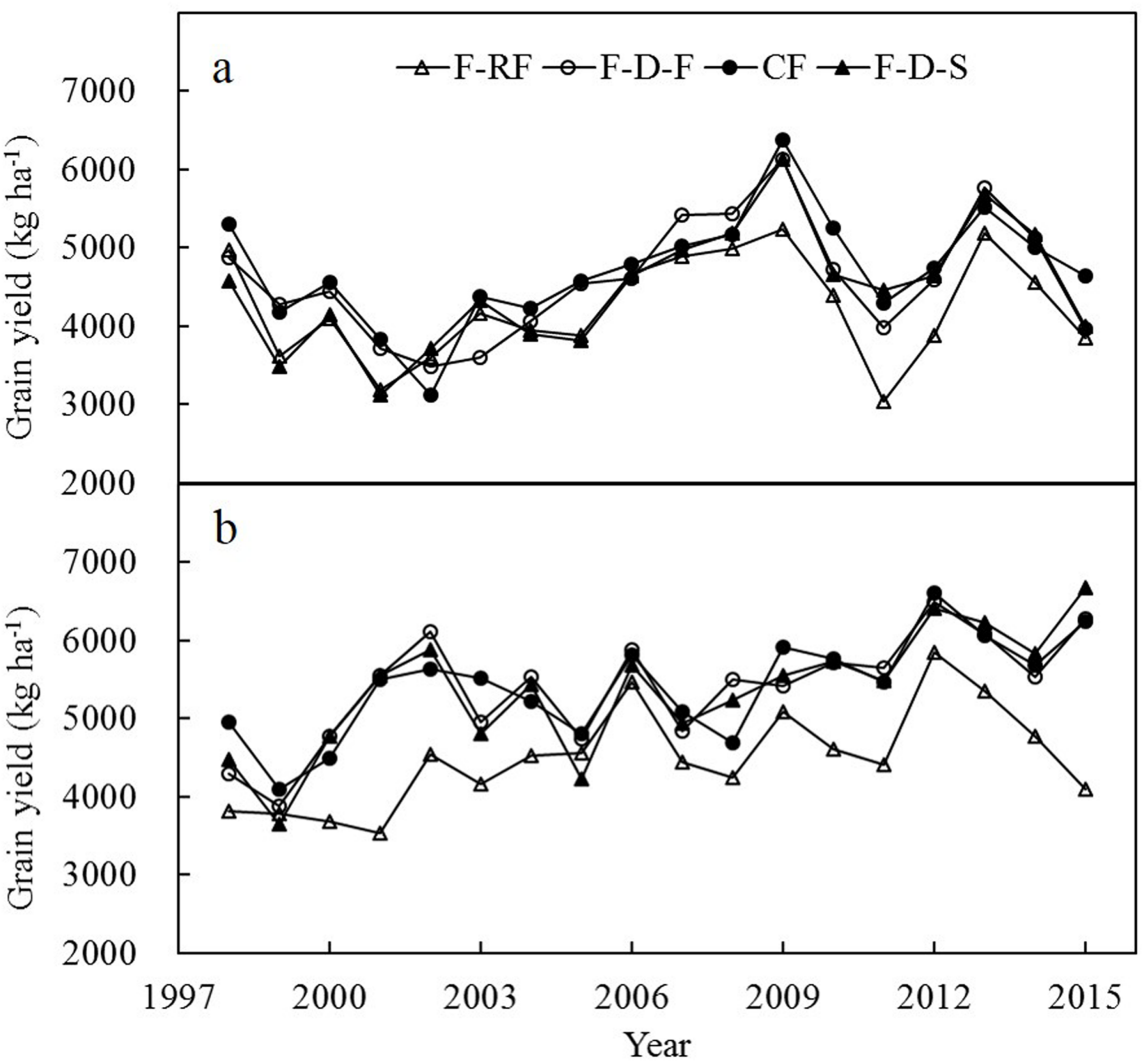
Rice grain yield in early-rice (a) and late-rice season (b) under different treatments. See Fig 2 for CF, F-D-F, F-D-S, and F-RF.

Rice grain yield under different modes of water management is presented in Fig 3 and Table 1. From 1998–2015, early-rice yield for F-D-F, F-D-S, CF, and F-RF, respectively, ranged from 3,600–6,123 kg ha^-1^, 3,120–6,135 kg ha^-1^, 3,120–6,380 kg ha^-1^, and 3,037–5,239 kg ha^-1^; and late-rice yield for F-D-F, F-D-S, CF, and F-RF, respectively, ranged from 3,885–5,848 kg ha^-1^, 3,653–6,667 kg ha^-1^, 4,103–6,615 kg ha^-1^, and 3,540–5,848 kg ha^-1^ (Fig 3). On average, the ranking of grain yield was CF > F-D-F > F-D-S > F-RF in early-rice season and CF ≥ F-D-F ≥ F-D-S > F-RF in late-rice season (Table 1). Compared with CF, F-D-F and F-D-S decreased grain yield by 2.6% and 5.2% in early-rice season, respectively. In contrast, in late-rice season, grain yields in CF, F-D-F, and F-D-S plots were almost same. Compared with CF, F-RF decreased grain yield by 10.4% in early-rice season and 17% in late-season.

### Water productivity

**Fig 4:**
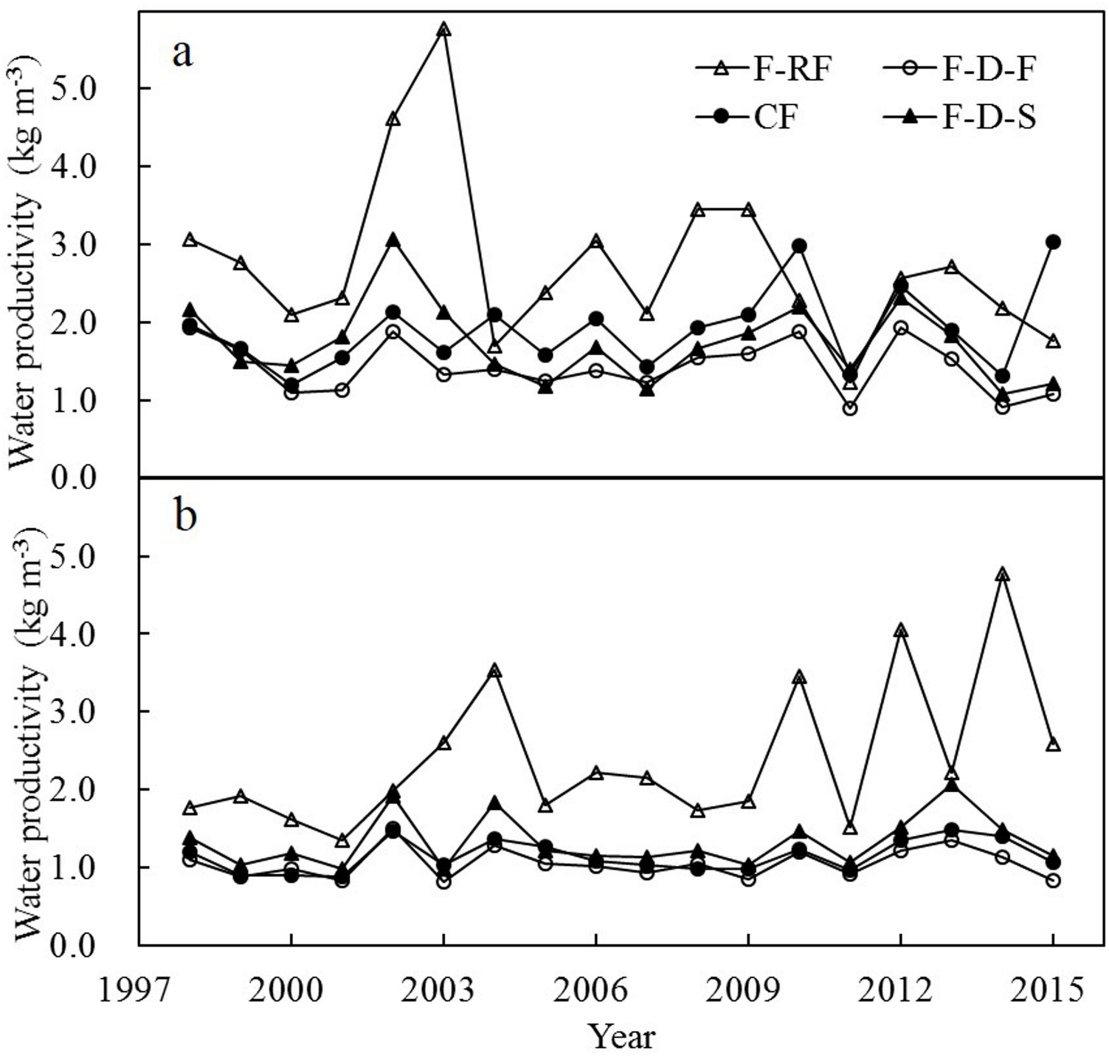
Water productivity in early-rice (a) and late-rice season (b) under different treatments. See Fig 2 for CF, F-D-F, F-D-S, and F-RF.

Water productivity (WP) under different modes of water management is presented in Fig 4 and Table 1. From 1998–2015, WP for F-D-F, F-D-S, CF, and F-RF, respectively, ranged from 0.89–1.94 kg m^-3^, 1.08–3.07 kg m^-3^, 1.19–3.03 kg m^-3^, and 1.23–5.78 kg m^-3^ in early-rice season; and ranged from 0.83–1.50 kg m^-3^, 0.98–1.48 kg m^-3^, 0.89–1.48 kg m^-3^, and 1.36–4.78 kg m^-3^ in late-rice season (Fig 4). On average, the ranking of WP was F-RF > CF > F-D-S > F-D-F in early-rice season and F-RF > F-D-S > CF > F-D-F in late-rice season (Table 1). The ranking of WP is quite inverse with that of irrigated water. The WP of F-RF was usually higher and varied more than that under the other three treatments. Contrast with F-D-F and F-D-S, in early-rice season, CF tended to increase WP. In late-rice season, CF, F-D-F, and F-D-S produced similar grain yields, however, F-D-S consumed less irrigation, and tended to increase WP. Compared with CF, F-D-F consumed more irrigated water, and still decreased grain yield, leading to WP decreased by 25% in early-rice season and by 8% in late-rice season. Compared with F-D-F, F-D-S saved more irrigated water with a small yield reduction, leading to an increase in WP by 22% in early-rice season and by 26% in late-rice season. Compared with CF, F-D-S decreased WP by 9% in early-rice season, however, increased WP by 16% in late-rice season. In addition, including irrigated water and precipitation, the total water productivity from 1998–2015 for F-D-F, F-D-S, CF, and F-RF, respectively, ranged from 0.34–0.79 (0.57 on average) kg m^-3^, 0.38–0.85 (0.60 on average) kg m^-3^, 0.32–0.92 (0.65 on average) kg m^-3^, and 0.40–1.01 (0.67 on average) kg m^-3^ in early-rice season; and ranged from 0.43–0.73 (0.59 on average) kg m^-3^, 0.44–0.87 (0.66 on average) kg m^-3^, 0.44–0.81 (0.62 on average) kg m^-3^, and 0.49–1.18 (0.79 on average) kg m^-3^ in late-rice season.

## Discussion

Rice is one of the largest users of the world’s fresh water resources. Evapotranspiration in flooded rice fields is 4–7 millimeters per day, slightly higher than that in aerobic fields [11, 15]. However, the percolation loss ranges from several to tens, or even hundreds of millimeters per day, depending on soil texture, age of rice cultivation, water depth, among others [2, 7, 15–17]. Belder et al. have reported that water consumption (precipitation plus irrigated water) was 600–900 mm under continuous flooding [7]. In the present study, CF consumed 263 mm irrigated water plus 483 mm precipitation in early-rice season and 484 mm irrigated water plus 397 mm precipitation in late-rice season.

It is generally accepted that, in paddy fields, irrigation with standing water would lead to more water losses from percolation and seepage [11–12, 16–19]. In the present study, F-D-S saved more irrigated water (61 mm in early-rice season and 106 mm in late-rice season) compared with F-D-F, which is consistent with the above conclusion. However in contrast with CF, F-D-S increased irrigated water in early-rice season, and F-D-F increased irrigated water in both early and late-rice seasons. This could be attributed to the following reasons. First, continuous flooding could improve plow sole structure that controlled infiltration rate [20]. As we can see in Fig 2, CF did not reduce irrigated water compared with F-D-F in the first several years of the long-term experiment. It is reported that the average infiltration rates for three paddy fields with a cultivation duration of 3, 20, and 100 years were 28.0, 0.79, and 0.16 cm per day, respectively, demonstrating a strong dependence of the infiltration rate on the age of the field [17]. Second, soil drying may lead to shrinkage and cracking, thereby risking increased soil water loss [2, 16, 18]. Finally, CF collected rainwater in fallow season, which mitigated soil cracking and reduced water requirement for land preparation.

More than 75% of Asian rice production occurs in irrigated areas, which occupies about 55% of total rice area in this region [21]. Many studies have shown that continuous flooding in the field is not essential to achieve high grain yield in rice. Compared with continuous flooding irrigation, both alternate wetting and drying irrigation and saturated irrigation could increase or maintain grain yield if minimum water potential of the soil was controlled reasonably according to soil properties and varieties [7, 18, 22–28]. In the present study, compared with CF, F-D-F and F-D-S maintained rice yields in late-rice season, but tended to decrease rice yields in early-rice season (Fig 3 and Table 1). During early stage of early-rice season, the temperature was relatively low (at around 20°C). The omission of a floodwater layer can expose the rice plant’s meristems to temperature extremes and thus affect plant growth [29]. In consistence with previous studies, grain yield decline was observed under F-RF, and the reduction was greater in dry season (late-rice season) than in wet season (early-rice season) (Fig 3 and Table 1), indicating unstable production under F-RF.

Agricultural water productivity (WP) is an important indicator of agricultural water management. Bouman and Tuong analyzed multi-site data and concluded that WP, including rainwater and irrigated water, in continuous flooded rice was typically 0.2–0.4 kg m^-3^ in India and 0.3–1.1 kg m^-3^ in the Philippines [2]. In the present study, the WP, including rainwater and irrigated water, in CF was 0.32–0.92 kgm^-3^ for early-rice and 0.44–0.81 kgm^-3^ for late-rice. High WP value with significant yield reduction carries less interest especially when food supply is not enough. In the present study, F-RF caused large grain yield reduction, although it increased WP. Some previous studies have demonstrated that saturated soil culture can not only save water but also maintain or even increase yields [2, 23, 28]. In the present study, there was little difference in yields between F-D-F and F-D-S, but F-D-S consumed less irrigation, leading to higher WP. In contrast, CF increased both grain yield and WP compared with F-D-F.

Compared with continuous flooding, various modes of water-saving management have one thing in common: shortening soil flooded period. However, flooded conditions are set not only for growth of rice plants but also as a management tool. For example, flooded condition is beneficial for soil puddling which could reduce water loss from percolation and seepage [16], and flooded condition can suppress germination of weed. So, flooded condition in early stage is necessary to make rice cultivation easy and efficient. In the present study, CF consumed less water compared with F-D-F, suggesting that continuous flooding could improve plow sole structure to reduce percolation. In practice, excellent water management strategies should use rainwater efficiently, such as to collect rainwater before land preparation, to reduce surface runoff by high ridge in case of prospective drought, to puddle soil sufficiently to reduce percolation and seepage losses, and to avoid drainage unless necessary. These measures are important for paddies without sufficient irrigation. Besides, considering constraints of labor, it should avoid frequent irrigation. For example, non-flooded irrigation is laborious and time-consuming. It is not cost-efficient for smallholders to transport and install pump machine too often. In contrast, the cycle of alternate flooded and non-flooded is longer. In addition, it is important to note that the fields should be irrigated before plant photosynthesis is disturbed or before soil crack appears [18, 28].

## Conclusions

This paper examined differences in water consumption, rice yields, and water productivity under different water management models in double rice systems in a long-term field experiment in south China, a major rice-producing area of in China. F-RF consumed the least irrigated water with the lowest grain yield, which carried little interest. Compared with CF, F-D-F consumed more irrigated water, which still decreased grain yield, leading to a decrease in water productivity by 25% in early-rice season and by 8% in late-rice season. Compared with F-D-F, F-D-S saved much irrigated water with a small yield reduction, leading to an increase in water productivity by 22% in early-rice season and by 26% in late-rice season. The results indicate that CF is best for early-rice and FDS is best for late-rice in terms of grain yield and water productivity.
